# Supplementary material for: Suppressing peatland methane production by electron snorkeling through pyrogenic carbon in controlled laboratory incubations
Source: Nat Commun. 2021 Jul 5;12:4119. doi: 10.1038/s41467-021-24350-y (PMC8257765; doi:10.1038/s41467-021-24350-y)
Supplement: Supplementary file 1 — Supplementary Information [file 41467_2021_24350_MOESM1_ESM.pdf]

Supplementary Information

for

Suppressing peatland methane production by electron snorkeling through pyrogenic carbon in controlled laboratory incubations

Tianran Sun[1,2], Juan J. L. Guzman[3], James D. Seward[4], Akio Enders[1], Joseph B. Yavitt[5], Johannes Lehmann[1,6], and Largus T. Angenent*[2,3,6]

[1]Soil and Crop Sciences, School of Integrative Plant Science, College of Agriculture and Life Sciences, Cornell University, Ithaca, New York 14853, United States

[2]Center for Applied Geosciences, University of Tübingen, Tübingen 72074, Germany

[3]Department of Biological and Environmental Engineering, College of Agriculture and Life Sciences, Cornell University, Ithaca, New York 14853, United States

[4]Vale Living with Lakes Centre and the Department of Biology, Laurentian University, Sudbury, Ontario P3E 2C6, Canada

[5]Department of Natural Resources, Cornell University, Ithaca, New York 14853, United States

[6]Atkinson Center for a Sustainable Future, Cornell University, Ithaca, New York 14583, United States

* Corresponding author: l.angenent@uni-tuebingen.de

Number of pages: 33
Number of methods: 7
Number of figures: 14
Number of tables: 2

Supplementary Methods

Method S1: Peat soil samples

The peat soil samples were collected in June at the Mclean Bog located in Dryden, New York (42°30’ N, 76°30’ W). Soils were sampled from the surface and reached a depth of approximately 10-15 cm. McLean Bog is an ombrotrophic (= rain fed) peat site, which has an area of 0.004-km^2. Total peat depth is 8 m. *Sphagnum* species include *S. angustifolium* and *S. magellanicum*. Shrubs include *C. calyculata* and *V. corymbosum* (highbush blueberry). The sedge *Eriophorum vaginatum* (cotton sedge) is also commonly distributed. Further descriptions can be found in our previous studies[1-3].

Microbial Sequencing Analysis. For community sequencing, DNA was extracted from peat samples with MoBio (now QIAGEN) Laboratories PowerSoil® DNA Isolation Kit and cleaned using the PowerClean® kit, following the manufacturers protocol with a heating step (65 °C for 30 minutes) added during the DNA extraction following bead beating. Sequences were collected on the MiSeq platform by the Department of Energy’s (DOE) Joint Genome Institute (JGI) for bacteria and archaea using the V4 region of SSU rRNA (515/806) primer pairing (forward primer 515F (5’-GTGCCAGCMGCCGCGGTAA-3’) and reverse primer 806R (5’-GGACTACHVGGGTWTCTAAT-3’)). Raw sequence data was obtained from the JGI database and sequences were first quality filtered using BBMap package[4]. PANDAseq version 2.1[5] was used to align forward and reverse reads, and aligned sequences were then processed with QIIME version 1.9.1[6], and USEARCH version 8[7] software using a 97% confidence value for OTU assignment[8,9]. Contigs were generated from complementary forward and reverse sequences while discarding sequence reads shorter than 240 base pairs (bp) or longer than 350 bp. The Greengenes 2013 database[10] was used for taxonomic assignment of bacterial and archaeal assemblages. Taxonomic identification data was generated and visualized using QIIME 1 scripts[6]. Microbial community structure of the peat soil was given in **Figure S1**.

Alternative terminal electron acceptors in the peat soil. The average production ratio of CO_2 to CH_4 dropped from 15 to 8 during the 9 days of incubation period (**Figure S2**), which was comparable to the ratios (range 1:1 to 40:1) reported by previous studies on several northern peat soils[3,11-13]. Higher CO_2 production than CH_4 indicated that alternative terminal electron acceptors

(other than O_2) existed in the anaerobic peat soil. Iron is a common alternative electron acceptor found in peat soils and contributes to alternative respiration not only influencing the mitigation of methanogenesis[14,15] but also the anoxic methane oxidation[16]. The peat soil used in this study, however, was from an ombrotrophic site which contains no inlet or outlet streams so that the iron inputs are usually insignificant. The measured iron concentration in the peat porewater is 4.2±1.3 μmol L^{-1} at 0-40 cm depth and 1.8±0.1 μmol L^{-1} at 100 cm depth. It has also been reported that the iron only contributed less than 2% to the total electron exchange capacities of ombrotrophic peat soils[17]. In comparison, the high carbon content (52%, dry weight percentage) in the peat soil indicated that organic matter, particularly its quinone moieties[18], was the major alternative terminal electron acceptor. Organic matter has been widely studied and demonstrated its importance in accepting electrons in anoxic soil conditions[19,20], especially under long-term reducing conditions after the exhaustion of such as NO_3^-, iron and manganese phases, and SO_4^{2-} ref[3]. The highest number of electron acceptance in the peat soil was determined at 785±120 μmol e^- g^{-1} soil carbon in the bioelectrochemical peat-soil incubations **(Figure 1d** in the main text), which was close to the reported electron accepting capacities of organic matters (a few hundred to thousand μmol e^- g^{-1} soil carbon) of several northern peat soils[17,21,22]. Another reason for the higher CO_2 production could be the introduction of oxygen into the peat soil during sampling. Even though we transferred the soil into sample bags as quickly as we could, a small amount of oxygen penetration into the peat soil was inevitable.

Method S2: Peat-soil incubation preparation

We used two layers of Ziplock bags to store each pack of soil samples to prevent oxygen penetration during transportation. The soils were stored in the lab at room temperature and dark environment until the incubation started. For the incubation, around 200 grams of soil was picked in the middle of each sampling pack, which was then homogeneously mixed to make one stock soil sample. The stock soil sample has a water content of 90% and a pH of 4.1. Afterwards, we divided the stock soil sample into three 10-g portions as the triplicates of one set of incubation. Each 10-g portion was individually placed into a 50 mL beaker and submerged with DI water to 30 mL for a full suspension. The beaker was pre-fixed in a 180 mL jar using hot glue. The jar was equipped with a gas-tight lid that had several rubber septa for gas sampling and pyrogenic carbon addition (**Figure S3a** and **b**). 50 mL DI water was added in the jar (surrounding the beaker to keep 100% moisture), which left the gas phase volume of each jar at 130 mL. The above-described preparation process was repeated for each individual incubation until fulfilling the designed incubation purposes. All preparations were performed in an anaerobic box with a continuous flow of N_2 gas. The closed jar was fully flushed once again with N_2 gas immediately prior the start of the incubation. A magnetic stir bar (400 rpm) was used in the soil suspension to maintain homogenous during the incubation. The total duration was 9 days for all peat-soil incubation. This incubation duration was set based on soil respiration rate. We found that after 9 days of incubation, the CO_2 production rate had dropped to 40% of the initial rate and reached a slow decrease phase (**Figure S2**). This result indicated that available nutrient and substrate in the peat soil had been largely depleted and soil respiration was approaching a pseudo-equilibrium condition.

Method S3: Microcosm peat-soil incubation

The effect of redox-cycling electron transfer of the pyrogenic carbon functional groups on electron snorkeling was investigated in microcosm peat-soil incubations (**Figure S3a**). Pyrogenic carbon produced at 400-500°C was used to study the redox-cycling electron transfer, due to its enrichment of functional groups (oxygen:carbon and hydrogen:carbon ratios range from 0.15 to 0.11 and 0.6 to 0.4, respectively). In contrast, the oxygen:carbon and hydrogen:carbon ratios of the pyrogenic carbon produced at 800°C are only 0.06 and 0.14[23], which indicated a condensed carbon structure and lack of functional groups. Further, for pyrogenic carbon produced at 500°C, we found that the redox-cycling electron transfer rate (0.16 mmol e^- g^{-1} pyrogenic carbon day^{-1}, **Figure 3a** in the main text) was about 1000 times faster than the conductive electron transfer rate (0.7 µmol e^- g^{-1} pyrogenic carbon day^{-1}, **Figure 3c** in the main text). No capacitive electron transfer process was detected in the pyrogenic carbon produced at 500°C (**Figure 3b** in the main text). Therefore, any electron snorkeling process occurred through the pyrogenic carbon that was produced in this low temperature range was mainly a result of the redox-cycling electron transfer of the functional groups. The microcosm peat-soil incubations were carried out with the soil native microbiota (i.e., without any further inoculation or nutrient addition) and no autoclavation was performed. The incubation temperature was maintained at 32°C in an incubation room. Lights were off all the time except for sampling. The gas phase was measured once a day by the Picarro stable isotope analyzer (G2201-I, Santa Clara, CA, USA). After each measurement, the gas phase of incubation was completely replaced by nitrogen gas.

The driving force of redox-cycling electron transfer was dependent on the inherent reduction potential of the functional groups (-0.2 to +0.25 V for quinone/hydroquinone couple[24-27]), which snorkeled extracellular electrons by spontaneous electron accepting (from alternative respiration) and donating (to alternative terminal electron acceptor) cycles. The number of snorkeled electrons by the redox-cycling electron transfer was quantified by the electron accumulation in the peat soil (i.e., the electrons that were accepted by alternative terminal electron acceptor (soil organic matter, see **Method S1**) and hold in the peat soil other than lost in the form of CH_4 emission). Electron accumulation was quantified by the increased electron donating capacity of the peat soil, using a previously reported hydrodynamic cyclic voltammetric method[28]. Briefly, 1 mL soil suspension was sampled out at day 1, 3, and 9 of each treatment and mixed with a 54 mL ferricyanide solution.

This total 55 mL mixture contained 0.36 g dry soil carbon L^{-1} solution, 10 mM potassium ferricyanide, and 3 M NaCl as the supporting electrolyte. After overnight shaking and reacting, a cyclic voltammetry was performed in the mixture using a glassy-carbon rotating-disk electrode (6 mm diameter). Due to the reduction of ferricyanide and production of ferrocyanide, an increased oxidation current (j_{Ferro}, A cm^{-2}) appeared in the hydrodynamic cyclic voltammograms (**Figure S4**). The electron donating capacity of the peat soil was quantified by measuring the final production of ferrocyanide using eq. S1 and S2:

$$Q_{ED} = \frac{nV[\mathrm{Ferro}]}{m} \quad \text{eq. S1}$$

$$[\mathrm{Ferro}] = \frac{j_{\mathrm{Ferro}}}{0.62nFD_{Ferro}^{2/3}v^{-1/6}\omega^{1/2}} \quad \text{eq. S2}$$

in which Q_{ED} is the number of donated electrons from the peat soil (mol e^- g^{-1} soil carbon), which was projected to the number of accumulated electrons in the peat soil, n = 1 is the number of electrons exchanged per mol ferricyanide reduction, V is the volume of solution (55 cm^3), m is the mass of dry soil carbon (0.02 g), and the [Ferro] is the ferrocyanide concentration (mol cm^{-3}). F is the Faraday constant (96,485 C mol^{-1}), D_{Ferro} is the diffusion coefficient of ferrocyanide at 30°C, 4.27×10^{-6} cm^2 s^{-1} ref[28], ν is the kinematic viscosity of 3 M NaCl solution at 30 °C (9.83×10^{-3} cm^2 s^{-1}, ref[28]), ω is the RDE rotation speed (104.7 rad s^{-1}).

Method S4: Bioelectrochemical peat-soil incubation

The effect of capacitive and conductive electron transfer of the pyrogenic carbon matrices on electron snorkeling was investigated in bioelectrochemical peat-soil incubations. We implemented a bioelectrochemical circuit in a two-chamber bioelectrochemical system (**Figure S3b)** in which a pyrogenic carbon rod was the working electrode (WE) and placed in the peat soil chamber along with an Ag/AgCl (saturated KCl) reference electrode (RE). The graphite rod counter electrode (CE) was placed in a separated chamber to avoid any generation of hydrogen in the peat soil chamber, which could potentially enhance the methanogenesis and methane emission. The bioelectrochemical peat-soil incubations were carried out with the soil native microbiota (i.e., without any further inoculation or nutrient addition) and no autoclavation was performed. The incubation temperature was maintained at 32°C in an incubation room. Lights were off all the time except for sampling. The gas phase was measured once a day by the Picarro stable isotope analyzer (G2201-I, Santa Clara, CA, USA). After each measurement, the gas phase of incubation was completely replaced by nitrogen gas.

Capacitive electron transfer refers to an intermittent electron transfer process that is constituted by a series of electron storage and release cycles. The capacity of electron storage and release relies on the capacitance of pyrogenic carbon matrices. Due to the faster electron storage rate from microbes to the carbon matrices than electron release rate from the carbon matrices to SOM (**Figure S5**), extracellular electrons started to accumulate in the carbon matrices. We periodically discharged the accumulated electrons in the carbon matrices by applying a +0.5 V (vs. SHE) electrical potential on the pyrogenic carbon WE (depicted by the red dash line in **Figure S3b**). Discharge of pyrogenic carbon induced a passing of electric current through the bioelectrochemical circuit (**Figure S6**). By integrating the current as a function of incubation time, we quantified the number of accumulated electrons in the carbon matrices during capacitive electron transfer. Before each discharging period, no potential was applied on pyrogenic carbon, therefore the initial electron accumulation was spontaneous and relied on the activity of extracellular electron transfer microbes and the capacitance of pyrogenic carbon matrices. We initiated the discharging procedure after 3 days of preincubation to facilitate the adaptation of soil microbes to the carbon matrices. We also monitored the current signals of the first 3 days of incubation but did not observe any current increase as a result of extracellular electron transfer (the

solid red line in **Figure 1d** in the main text). This indicated that the activity of extracellular electron transfer microbes was not being considerably stimulated by the application of potential and the following growth of microbes was the result of capacitive election transfer supported microbial respiration (the red dots in **Figure 1d** in the main text). The discharging procedure was applied once every 11.5 h of capacitive electron transfer and each application period lasted for 0.5 h until the discharging current reached a steady state.

Conductive electron transfer refers to a continuous electron transfer process that derives from the conductivity of pyrogenic carbon matrices. For conductive electron transfer, we applied a constant +0.5 V (vs. SHE) electrical potential on the pyrogenic carbon WE to provide a sufficient driving force and investigate the conductive electron transfer under an accelerated electron accepting condition (depicted by the red solid line in **Figure S3b**). By eliminating the electron snorkeling limit induced by the terminal electron accepting step, we were able to overcome the electron accumulation as observed in capacitive electron transfer and target on resolving the controlling effect of only conductive electron transfer on the overall electron snorkeling process. Conductive electron transfer allowed a continuous acceptance of extracellular electrons by soil organic matter. Continuous transfer of electrons resulted in a passing of electric current through the bioelectrochemical circuit **(**blue line in **Figure 1d** in the main text). By integrating the current as a function of incubation time, we were able to quantify the number of accumulated electrons in soil organic matter through conductive electron transfer. Even though soil organic matter is electrode active, its activity on electrode is too low to generate featured current signals in response to applied potentials[29]. In our system, we confirmed this low activity phenomenon by performing cyclic voltammetric scans on pyrogenic carbon with the presence of soil organic matter (**Figure S5**). Results showed that no featured current peaks appeared either in oxidative or in reductive scan, indicating that the obtained current signals in bioelectrochemical peat-soil incubations were directly resulted from the capacitive and conductive electron transfers instead of soil organic matter mediated redox reactions.

Method S5: Calculation of the proportion of gases emitted from the respiration of pyrogenic carbon

Previous studies have shown that metabolism of pyrogenic carbon could modify the composition of soil microbial community[30], which potentially affect methanogenesis process[31]. Therefore, to further clarify the effect of biodegradation of pyrogenic carbon on methanogenesis activity, we performed isotopic experiments to investigate if and to what extent biodegradation of the pyrogenic carbon could contribute to overall gas emission. In all peat-soil incubations, the added pyrogenic carbon was labelled with ^{13}C, which resulted in a $\delta^{13}C$ of pyrogenic carbon at 774±2.3‰. Isotopic labelling was performed in growth chambers using ^{13}C labeled CO_2 as the source for shrub willow (i.e., the original biomass for pyrogenic carbon production) growth. Detailed labeling process and growth condition can be found in ref[23]. We monitored the $\delta^{13}CO_2$ and $\delta^{13}CH_4$ production during the microcosm (**Table S1**) and bioelectrochemical (**Table S2**) peat-soil incubations to track the respiration of pyrogenic carbon by the native peat-soil microbiota. The percentage of CO_2 and CH_4 (relative to the total gas emission from the peat soil) that were derived from the respiration of pyrogenic carbon were calculated based on eq. S3[23]:

$$P_{CO_2\ or\ CH_4} = \frac{\delta^{13}C_{PyC+soil} - \delta^{13}C_{soil}}{\delta^{13}C_{PyC} - \delta^{13}C_{soil}} \times 100 \qquad \text{eq. S3}$$

in which $P_{CO_2\ or\ CH_4}$ is the percentage of CO_2 or CH_4 that are derived from the respiration of pyrogenic carbon in the bioelectrochemical and microcosm peat-soil incubations, $\delta^{13}C_{PyC+soil}$ represents the $\delta^{13}C$ values of the peat-soil incubations with pyrogenic carbon, $\delta^{13}C_{soil}$ is the $\delta^{13}C$ values of the pyrogenic carbon-free control incubations (i.e., the peat soil only), $\delta^{13}C_{PyC}$ is the $\delta^{13}C$ value of the labelled pyrogenic carbon. The $\delta^{13}C$ values in terms "$\delta^{13}C_{PyC+soil}$" and "$\delta^{13}C_{soil}$" are either $\delta^{13}C$ of CO_2 or CH_4 as given in **Table S1** and **S2**.

Method S6: Microcosm pure-culture incubation

The microcosm pure-culture incubations were carried out in serum bottles at 32°C and ambient light condition (**Figure S3c**). Each serum bottle contained 50 mL growth media, which consisted of 2.5 g $NaHCO_3$, 0.25 g NH_4Cl, 0.52 g NaH_2PO_4, 1 g KCl, 1 mL of vitamin mix, 1 mL of mineral mix, and 40 mM sodium acetate per litter. *Geobacter sulfurreducen*s strain PCA (1 mL of stock culture at 0.1 OD) was inoculated as an alternative respiratory bacterium. Pyrogenic carbon produced at 500°C was added to each serum bottle to study its redox-cycling electron transfer in the microcosm pure-culture incubations. The addition rate of pyrogenic carbon was 1 mg pyrogenic carbon mL^{-1} growth media. Due to the enrichment of functional groups and less condensed carbon structure, the capacitive and conductive electron transfers through the carbon matrices that were produced at 500°C were highly diminished (green and orange lines in **Figure 3b** and **c** in the main text). Therefore, any electron snorkeling process occurred through the pyrogenic carbon that was produced in this low temperature was a result of the redox-cycling electron transfer of the functional groups.

During the incubation, pyrogenic carbon became more and more reduced due to the continuous accepting of electrons generated from *G. sulfurreducen*s respiration (i.e., the microbial reduction in eq. S4). Aliquots of the reduced pyrogenic carbon were sampled daily and re-oxidized by donating electrons to potassium ferricyanide (eq. S5).

$$\text{Acetate} + \text{PyC}_{\text{Ox}} \xrightarrow[\text{reduction}]{\text{Microbial}} \text{CO}_2 + \text{H}^+ + \text{PyC}_{\text{Red}} \qquad e^- \textit{ acceptance} \qquad \text{eq. S4}$$

$$\text{PyC}_{\text{Red}} + \text{Ferri} \xrightarrow[\text{oxidation}]{\text{Chemical}} \text{PyC}_{\text{Ox}} + \text{Ferro} \qquad e^- \textit{ donation} \qquad \text{eq. S5}$$

We determined the number of snorkeled electrons (Q, mol e^- g^{-1} pyrogenic carbon) of the complete redox cycle (eq. S4 + eq. S5) of pyrogenic carbon functional groups by measuring the final production of ferrocyanide (eq. S6[28]):

$$Q = \frac{nV[\text{Ferro}]_{\text{redox-cycling}}}{m} \qquad \text{eq. S6}$$

in which n = 1 is the number of electrons exchanged per mol ferricyanide reduction, V is the volume of solution (55 cm^3), m is the mass of pyrogenic carbon (0.01 g), and the $[Ferro]_{redox\text{-}cycling}$ is the ferrocyanide concentration (mol cm^{-3}) produced by the redox cycling of the functional groups.

The $[Ferro]_{redox\text{-}cycling}$ was measured by mixing 10 mL incubation solution with 45 mL potassium ferricyanide solution. This total 55 mL mixture contained 0.01 g pyrogenic carbon, 10 mM ferricyanide, and 3 M NaCl as the supporting electrolyte. After overnight shaking and reacting, a cyclic voltammetry was performed in the mixture using a glassy-carbon rotating-disk electrode (6 mm diameter). Due to the reduction of ferricyanide and production of ferrocyanide, an increased oxidation current (j_{total}, A cm^{-2}) appeared in the hydrodynamic cyclic voltammograms (**Figure S7**). After subtracting the background reduction induced oxidation current, we obtained the $[Ferro]_{redox\text{-}cycling}$ based on the Levich equation (eq. S7):

$$[Ferro]_{redox\text{-}cycling} = \frac{j_{total} - j_{inoculation} - j_{HQ}}{0.62nFD_{Ferro}^{2/3}v^{-1/6}\omega^{1/2}} \quad \text{eq. S7}$$

$$j_{inoculation} = 0.62nFD_{Ferro}^{2/3}v^{-1/6}\omega^{1/2}\ [Ferro]_{inoculation} \quad \text{eq. S8}$$

$$j_{HQ} = 0.62nFD_{Ferro}^{2/3}v^{-1/6}\omega^{1/2}\ [Ferro]_{HQ} \quad \text{eq. S9}$$

where F is the Faraday constant (96,485 C mol^{-1}), D_{Ferro} is the diffusion coefficient of ferrocyanide at 30°C, 4.27×10^{-6} cm^2 s^{-1} ref[28], ν is the kinematic viscosity of 3 M NaCl solution at 30 °C (9.83×10^{-3} cm^2 s^{-1}, ref[28]), ω is the RDE rotation speed (104.7 rad s^{-1}).

j_{total} in eq. S7 indicates the total oxidation current (**Figure S7c**), $j_{inoculation}$ (**Figure S7b**) and j_{HQ} (**Figure S7a**) in eq. S7 are the oxidation current induced by the background reduction. The background reduction derived from: (1) the immediate reduction of ferricyanide by the biofilm electrons after the inoculation of *G. sulfurreducen*s (i.e., eq. S8); and (2) the abiotic reduction of ferricyanide by the inherent hydroquinone groups in pyrogenic carbon (i.e., eq. S9). The $j_{inoculation}$ was assessed by inoculating microbes into the growth medium that contained sand but no pyrogenic carbon and other electron acceptors. $[Ferro]_{inoculation}$ indicates the concentration of ferrocyanide resulted from the ferricyanide reduction by biofilm electrons. The j_{HQ} was determined

in the growth medium with the addition of pyrogenic carbon but without microbe inoculation. $[Ferro]_{HQ}$ indicates the concentration of ferrocyanide resulted from the ferricyanide reduction by hydroquinone groups. By substituting the $[Ferro]_{redox\text{-}cycling}$ term with $[Ferro]_{HQ}$ in eq. 6, we determined the electron donation capacity (EDC) of pyrogenic carbon (produced at 500°C) at 0.125±0.02 mmol e^{-} g^{-1} pyrogenic carbon. By subtracting $j_{inoculation}$ and j_{HQ} from j_{total}, we obtained the oxidation current and more importantly the concentration of $[Ferro]_{redox\text{-}cycling}$. By introducing $[Ferro]_{redox\text{-}cycling}$ into eq. S6, we estimated the number (0.293±0.025 mmol e^{-} g^{-1} pyrogenic carbon) of transferred electrons during the redox-cycling electron transfer of pyrogenic carbon in supporting the growth of *G. sulfurreducen*s. In addition, due to the subtraction of background electrons that were donated from biofilm and hydroquinone groups, this number of transferred electrons during redox-cycling electron transfer also reflected the bioavailable electron accepting capacity (EAC) of pyrogenic carbon (produced at 500°C). The determined EDC and EAC values are in agreement with the previously reported results[32].

Method S7: Bioelectrochemical pure-culture incubation

We investigated the effect of the pyrolysis temperature on the electron snorkeling kinetics of capacitive and conductive electron transfers using the bioelectrochemical pure-culture incubations. A one-chamber bioelectrochemical system (**Figure S3d**), adapted from previously published studies[33,34], was used in the bioelectrochemical pure-culture incubations due to its autoclavable and easy-assemble features. All pyrogenic carbon WE, Ag/AgCl RE, and graphite CE electrodes were placed in the same chamber. The bioelectrochemical systems were autoclaved, filled with 15 mL of sterile growth media (consisted of 2.5 g $NaHCO_3$, 0.25 g NH_4Cl, 0.52 g NaH_2PO_4, 1 g KCl, 1 mL of vitamin mix, 1 mL of mineral mix, and 40 mM sodium acetate per litter), and placed in a 30°C water bath. N_2:CO_2 (80%:20%) gas was continuously sparged in the growth media to maintain anaerobic condition. *G. sulfurreducen*s strain PCA (1 mL of stock culture at 0.1 OD) was inoculated as an alternative respiratory bacterium in all pure-culture incubations.

Similar to bioelectrochemical peat-soil incubations (**Method S4**), an electrical potential (+0.5 V vs. SHE) was applied periodically (depicted by the red dash line in **Figure S3d**) on pyrogenic carbon to discharge the accumulated electrons in the carbon matrices during capacitive electron transfer. The potential was applied once every 11.5 h during the capacitive electron transfer and each application period lasted for 0.5 h until the discharging process reached a steady state. This low-frequency and short-period discharging regime was designed to avoid the formation of continuous electron transfer through the conductive electron transfer mechanism.

For conductive electron transfer, a range of constant potentials (+0.2 to +0.5 V vs. SHE) were applied (depicted by the red solid line in **Figure S3d**) on pyrogenic carbon to investigate the limiting effect of terminal electron accepting step on electron snorkeling of conductive electron transfer. The constantly applied potentials imposed similar effects to the reduction potentials of naturally occurring electron acceptors in accelerating electron accepting step from the carbon matrices. Therefore, by applying a range of low to high potentials on pyrogenic carbon, we successfully predicted the dependency of conductive electron transfer on the type and availability of environmental electron acceptors. This prediction has important implications in interpreting the function of conductive electron transfer in the presence of electron acceptors which possess high reduction potentials (such as Fe minerals and nitrate species) and can rapidly accept electrons out

of pyrogenic carbon through its conductivity. Pyrogenic carbon that was produced from low to high pyrolysis temperatures (400-800°C) was incubated in the bioelectrochemical pure-culture incubations. Both capacitive and conductive electron transfers induced passing of electric current through the bioelectrochemical circuit (**Figure 3b and c** in the main text and **Figure S8-S10**). By integrating the current as a function of incubation time, we quantified the number of snorkeled electrons in capacitive and conductive electron transfers.

Supplementary Figures

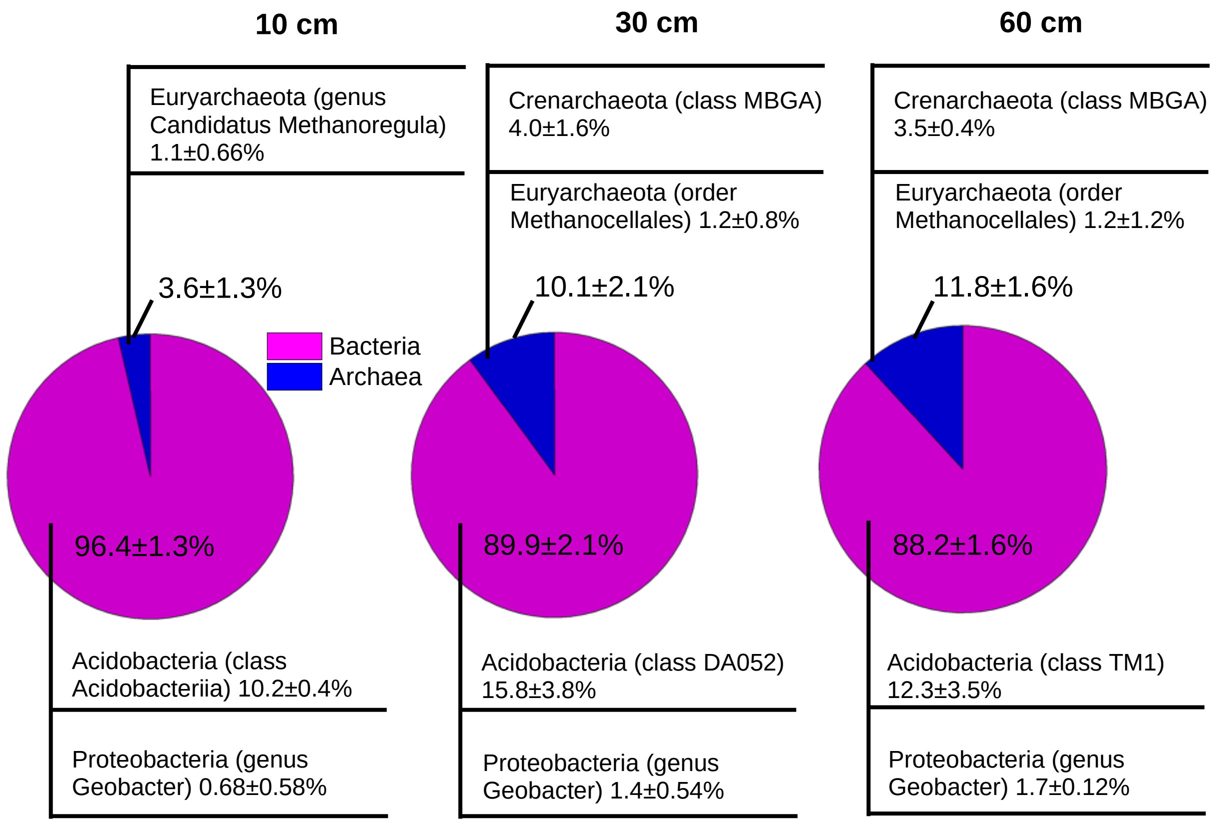

Figure S1 | Microbial community analysis of the studied peat soil at different soil depth (10-60 cm). The pie charts show the relative abundance of bacteria and archaea. The notes above the charts give the relative abundance of the most abundant archaea and methanogens in the archaea kingdom and the notes below the charts demonstrate the relative abundance of the most abundant bacteria and the relative abundance of *Geobacter* in the bacteria kingdom.

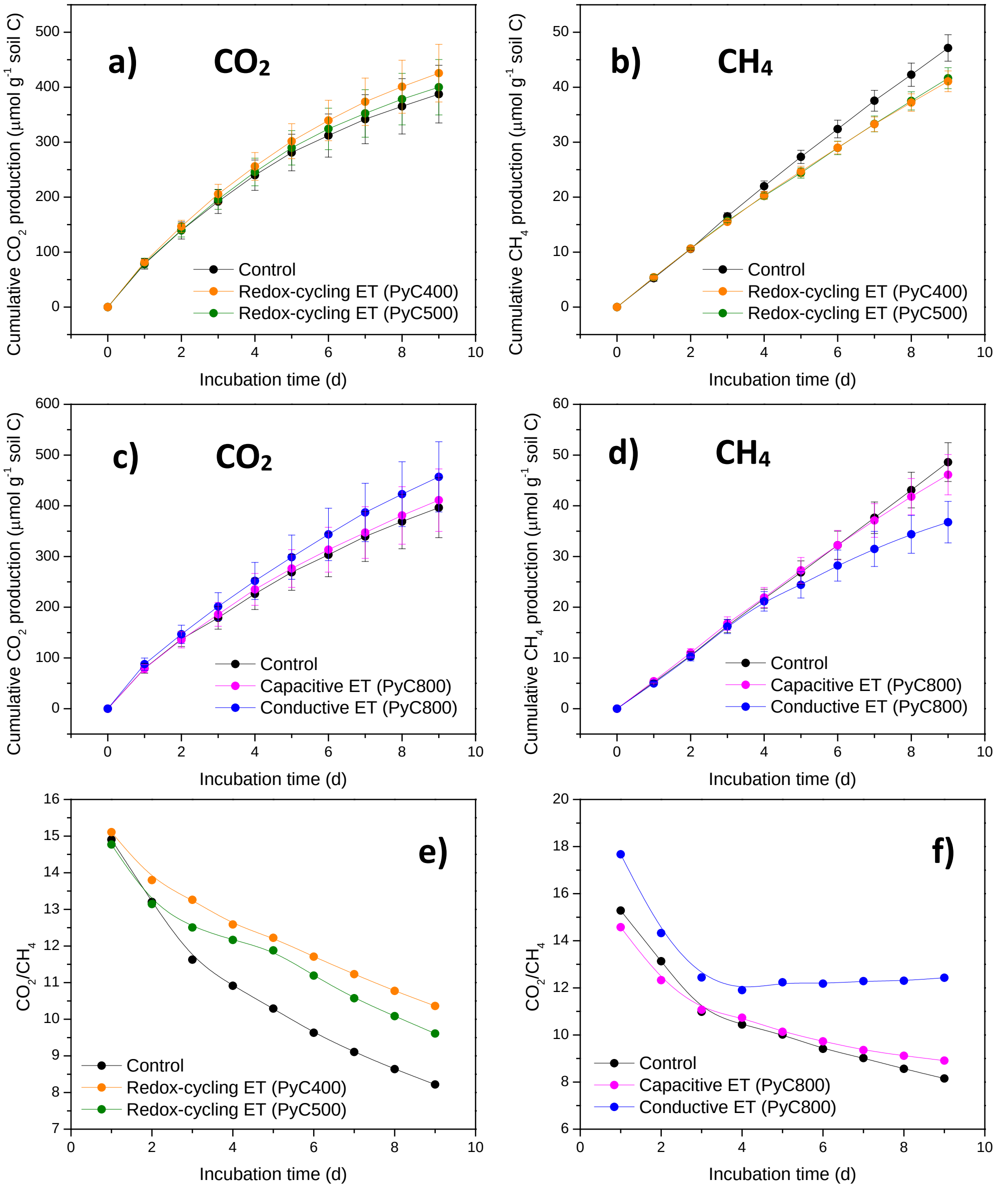

Figure S2 | Total gas emission from the peat soil (incubated in the dark at 32°C) as a function of incubation time. a and **b.** Total CO_2 and CH_4 emissions from the pyrogenic carbon-free control treatment and from the incubations that were associated with the redox-cycling electron transfer (ET) for electron snorkeling in the microcosm peat-soil incubations. **c** and **d.** Total CO_2 and CH_4 emissions from the pyrogenic carbon-free control treatment (i.e., the original peat soil emission) and from the incubations that were associated with capacitive and conductive ET for electron snorkeling in the bioelectrochemical peat-soil incubations. Error bars in **a-d** are s.d. of triplicate measurements (N=3). Data are presented as mean values +/- s.d. **e** and **f.** CO_2 to CH_4 ratios in the pyrogenic carbon-free control treatment and microcosm and bioelectrochemical peat-soil incubations. In all charts, PyC stands for pyrogenic carbon and the following numbers indicate the pyrolysis temperatures.

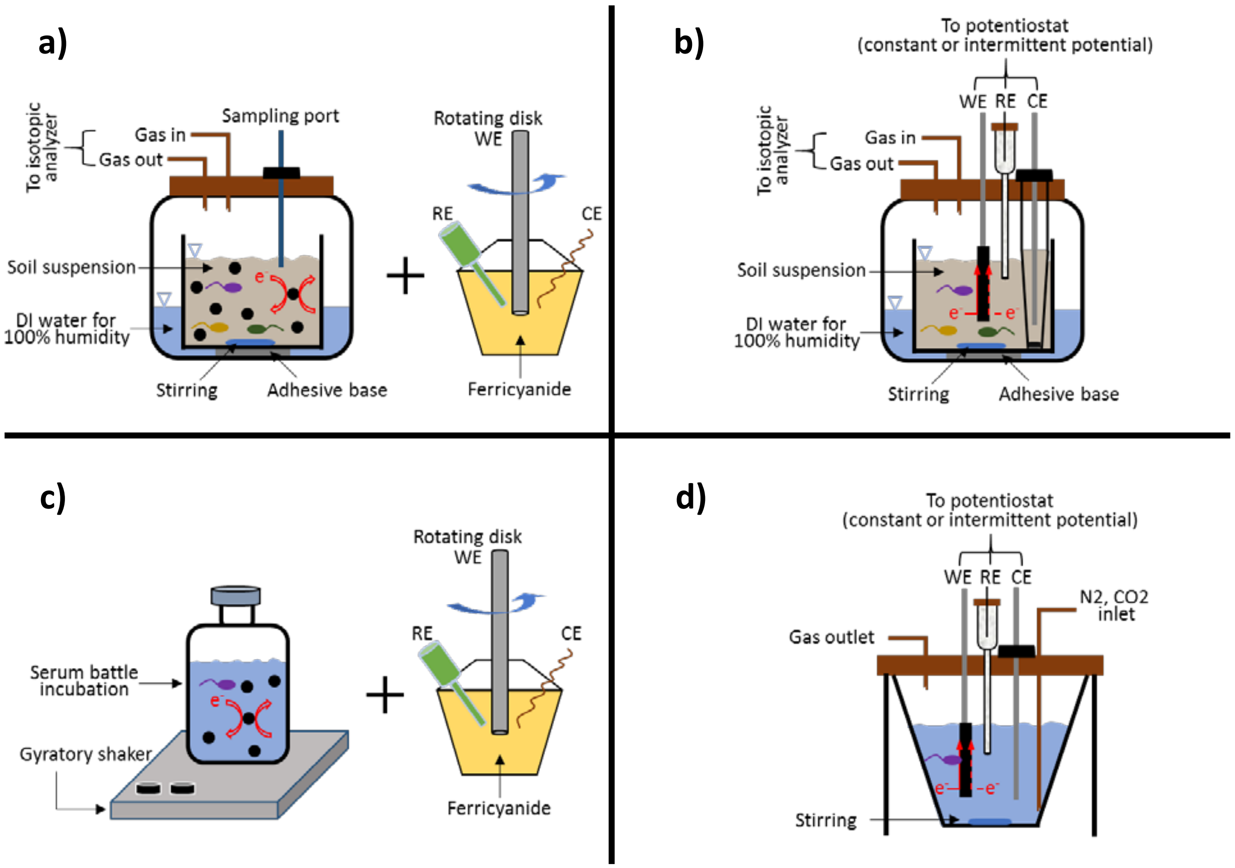

Figure S3 | Incubation setups. a. microcosm peat-soil incubation; **b.** bioelectrochemical peat-soil incubation; **c.** microcosm pure-culture incubation; and **d.** bioelectrochemical pure-culture incubation. WE, CE and RE in the figure indicate working electrode, counter electrode and reference electrode, respectively. All incubations were performed within an N_2 atmosphere. Figure S3 was created by authors.

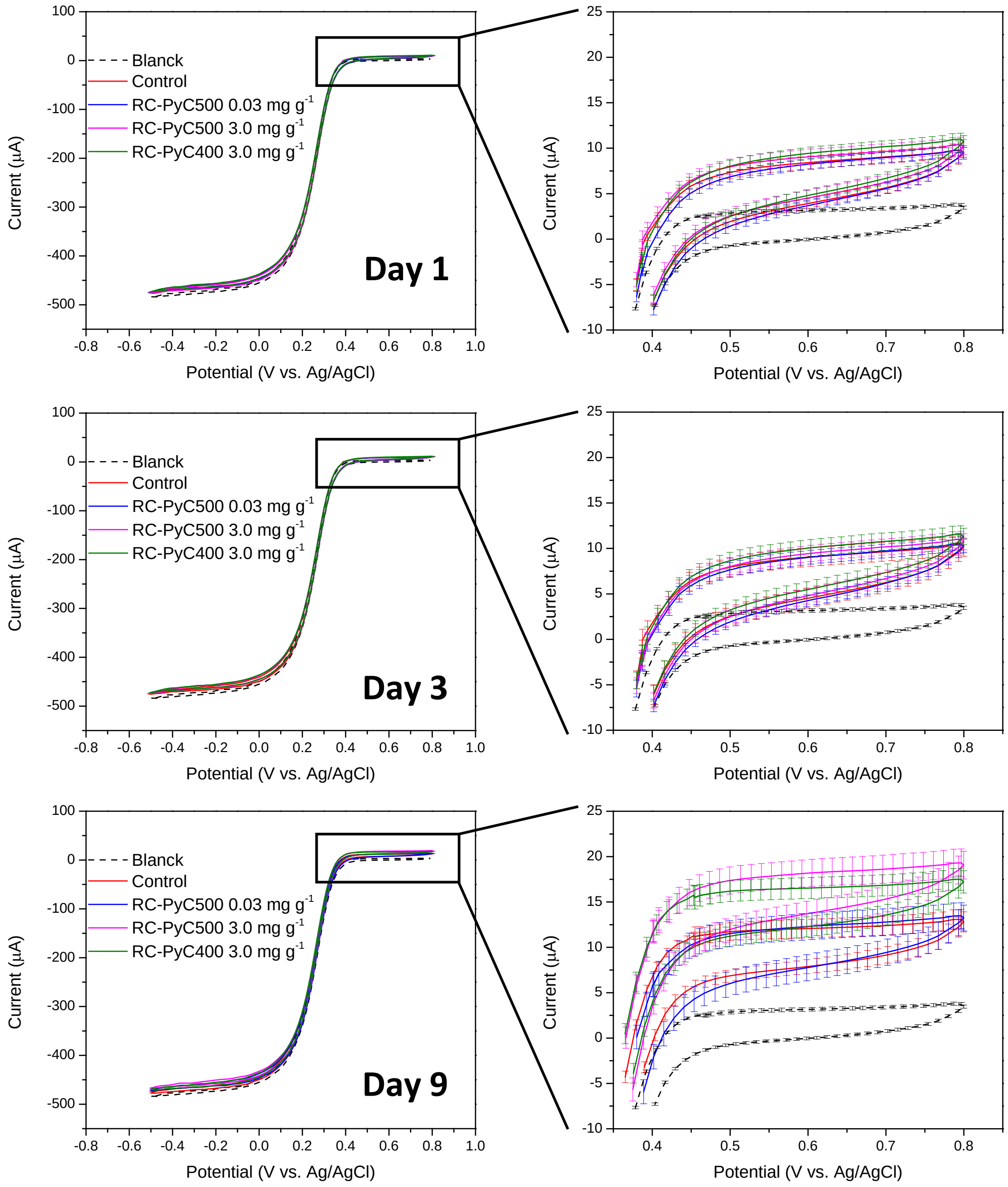

Figure S4 | Cyclic voltammograms (CVs) of the ferricyanide that was reduced by the peat soil in the microcosm peat-soil incubation (32°C and dark). Based on the oxidation current increase of ferricyanide (enlarged in the right charts), the number of donated electrons from the peat soil was determined and used to calculate the number of accumulated electrons in the peat soil induced by the redox-cycling electron transfer of the pyrogenic carbon functional groups. In the figure legend, blank indicates the CV of pure ferricyanide without reacting with the peat soil. Control indicates the CV of ferricyanide that was reduced by the peat soil only. RC-PyC500 and RC-PyC400 stand for the CV of ferricyanide that was reduced by the peat soil with the redox-cycling electron transfer (RC) of the pyrogenic carbon (PyC) produced at 500 and 400°C, respectively. The numbers are the application rate of the pyrogenic carbon in the peat soil. Error bars are s.d. of triplicate measurements (N=3). Data are presented as mean values +/- s.d.

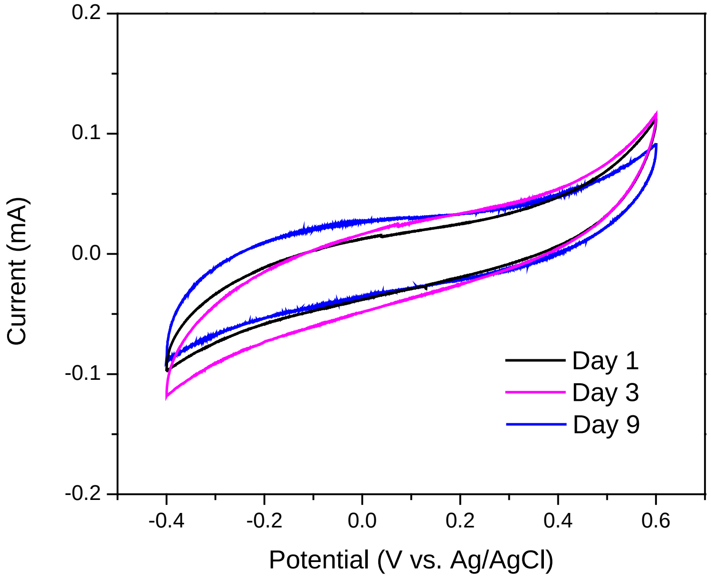

Figure S5 | Cyclic voltammetric scan of pyrogenic carbon (produced at 800°C pyrolysis temperature) in the peat soil of the bioelectrochemical peat-soil incubation (32°C and dark). Results showed that there was no featured peak current appeared over a 1 V scanning potential. Despite high conductivity of the pyrogenic carbon that were produced at high pyrolysis temperature[24] and strong redox activity of soil organic matter[20], lack of peak current indicated a slow electron transfer kinetics[35] across the carbon matrices and soil organic matter interface.

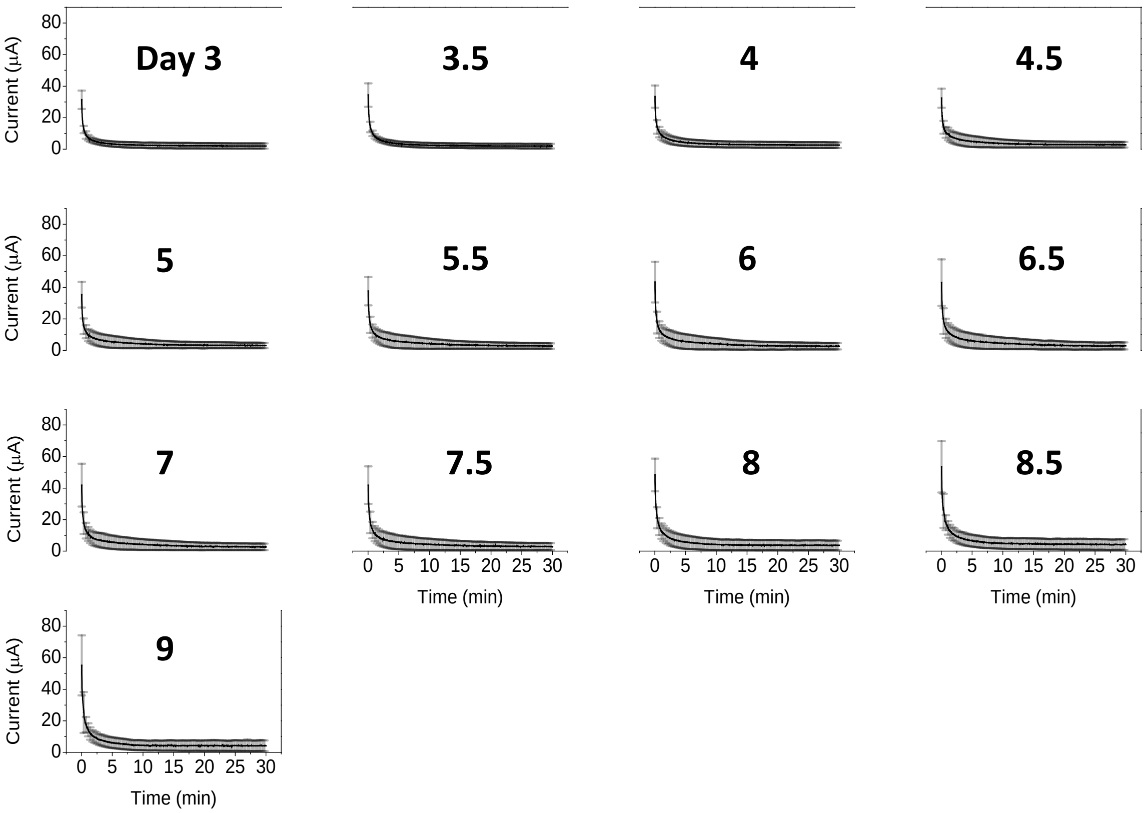

Figure S6 | Full discharging current profile of the capacitive electron transfer through the pyrogenic carbon matrices during the bioelectrochemical peat-soil incubation (32°C and dark). The capacitive electron transfer started after 3 days of pre-incubation to facilitate the adaptation of soil microbes to the carbon matrices. The discharging current was recorded every half a day and the discharging duration was 30 min. The recording frequency of the discharging current was 0.1 s^{-1}. Error bars are s.d. of triplicate measurements (N=3). Data are presented as mean values +/- s.d.

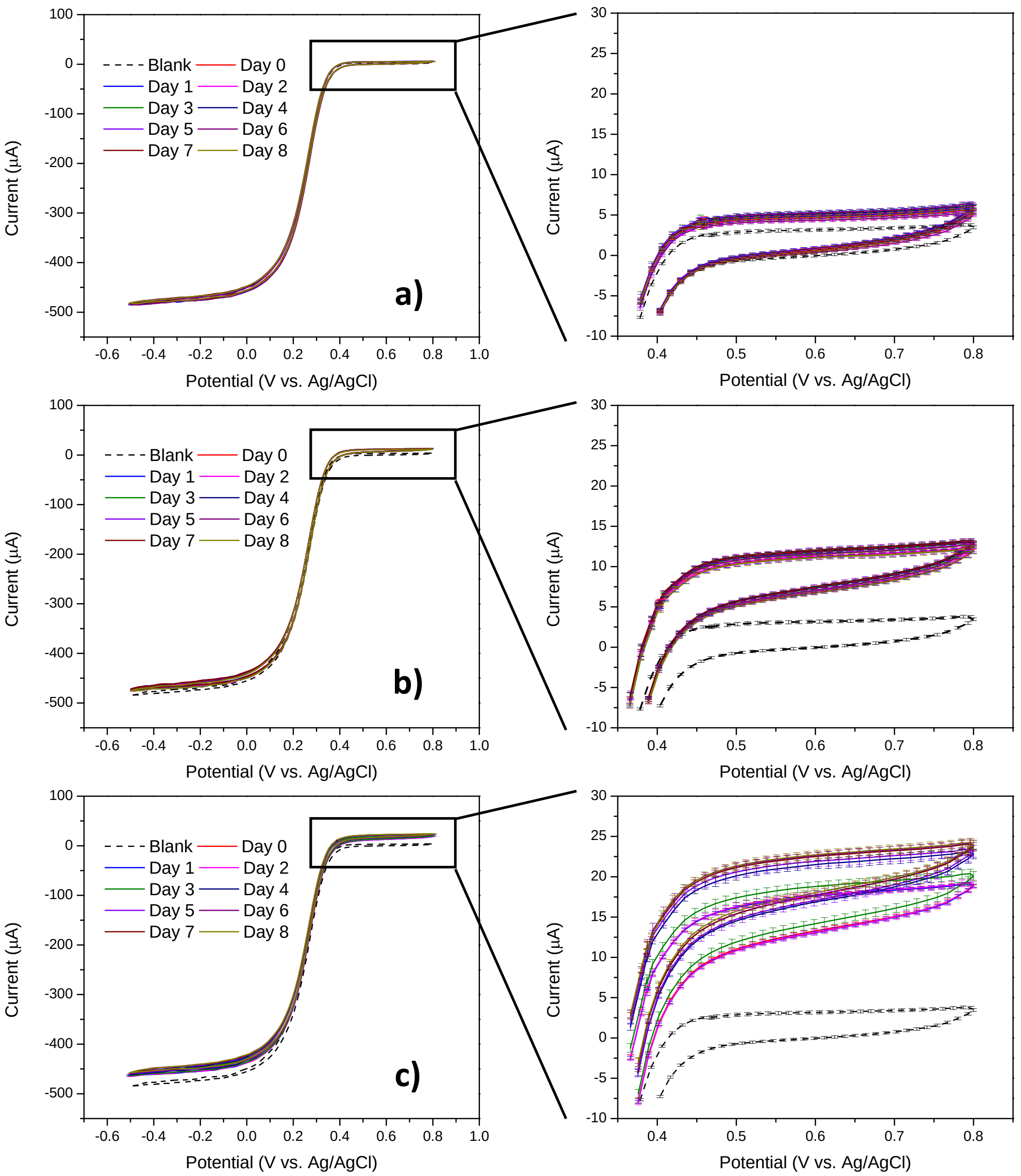

Figure S7 | Cyclic voltammograms (CVs) of the ferricyanide that was reduced during the microcosm pure-culture incubations (32°C and ambient light). The oxidation current (enlarged in the right charts) shown in **a** and **b** represents the background reduction of the ferricyanide by only pyrogenic carbon and *G. sulfurreducens*, respectively. In the pyrogenic carbon and *G. sulfurreducens* co-existed incubation (**c**), as microbes growing, surface functional groups started to accept electrons and the pyrogenic carbon became more and more reduced. The reduced pyrogenic carbon was then re-oxidized by donating electrons to oxidizer ferricyanide, which completed the redox cycling and led to an increase of oxidation current in the CVs of ferricyanide. Error bars are s.d. of triplicate measurements (N=3). Data are presented as mean values +/- s.d. The number of transferred electrons shown in **Figure 3a** in the main text was calculated based on the oxidation current in **c** by subtracting the background oxidation current of **a** and **b**.

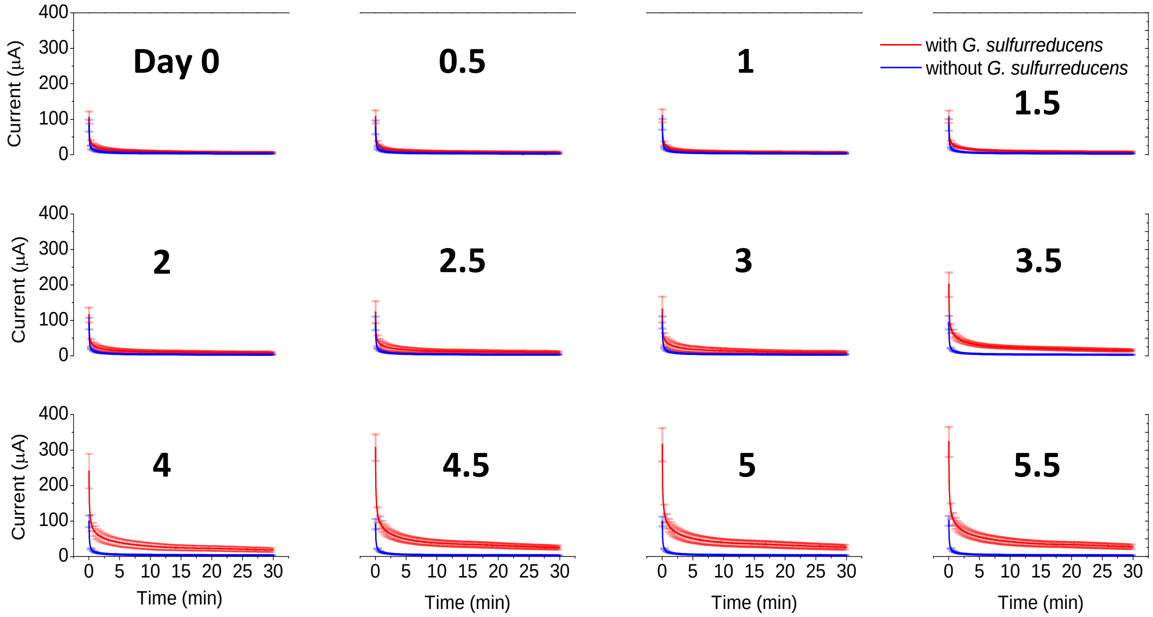

Figure S8 | Full discharging current profile of the capacitive electron transfer through the pyrogenic carbon matrices that was produced at 800°C during the bioelectrochemical pure-culture incubation (32°C and ambient light). A control incubation without *G. sulfurreduccens* was also performed to confirm that the increased discharging current through the carbon matrices was a result of the alternative respiration of *G. sulfurreduccens* by using the capacitive electron transfer as an electron snorkeling mechanism. The discharging current was recorded every half a day and the discharging duration was 30 min. The recording frequency of the discharging current was 0.1 s^{-1}. Error bars are s.d. of triplicate measurements (N=3). Data are presented as mean values +/- s.d.

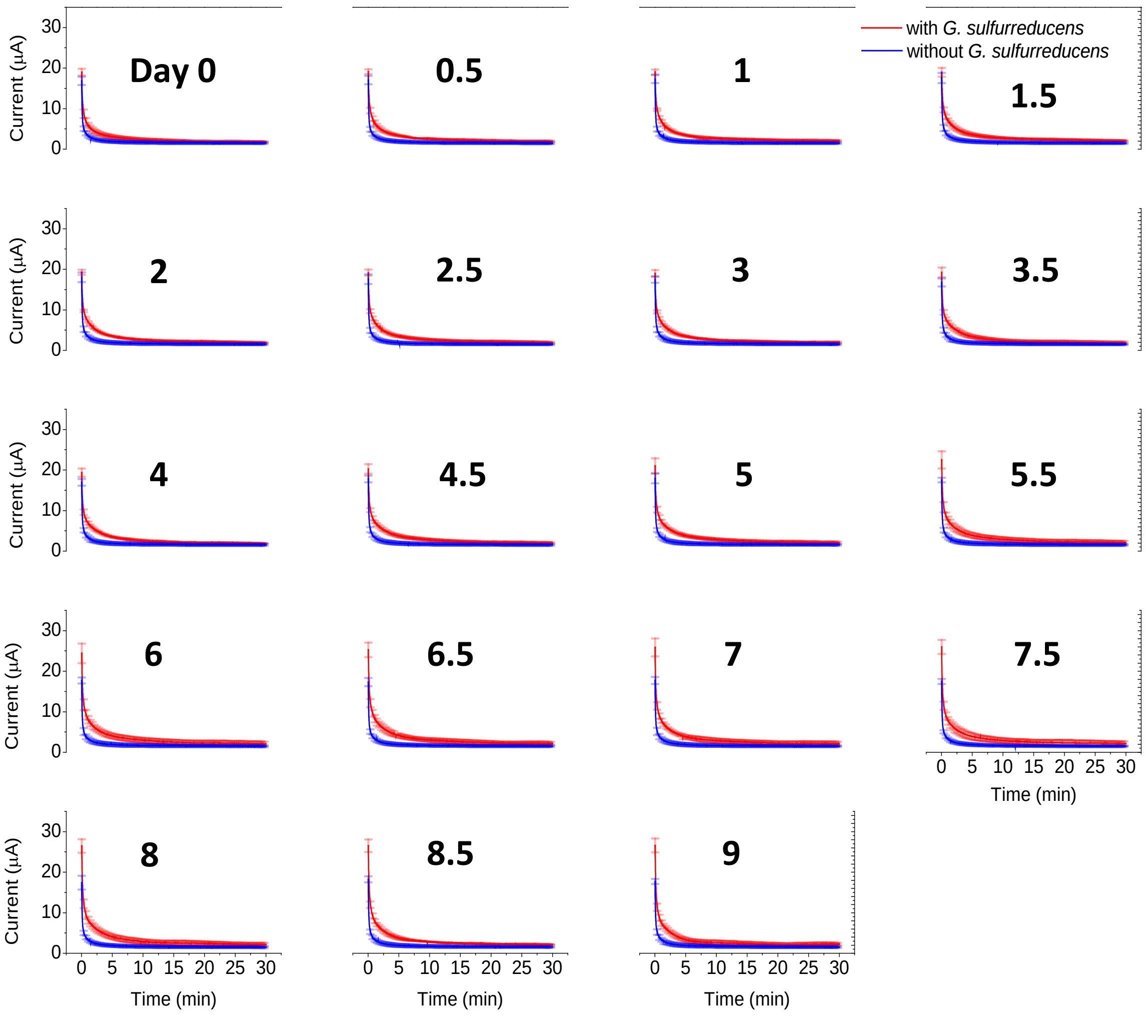

Figure S9 | Full discharging current profile of the capacitive electron transfer through the pyrogenic carbon matrices that was produced at 650°C during the bioelectrochemical pure-culture incubation (32°C and ambient light). A control incubation without *G. sulfurreduccens* was also performed to confirm that the increased discharging current through the carbon matrices was a result of the alternative respiration of *G. sulfurreduccens* by using the capacitive electron transfer as an electron snorkeling mechanism. The discharging current was recorded every half a day and the discharging duration was 30 min. The recording frequency of the discharging current was 0.1 s^{-1}. Error bars are s.d. of triplicate measurements (N=3). Data are presented as mean values +/- s.d.

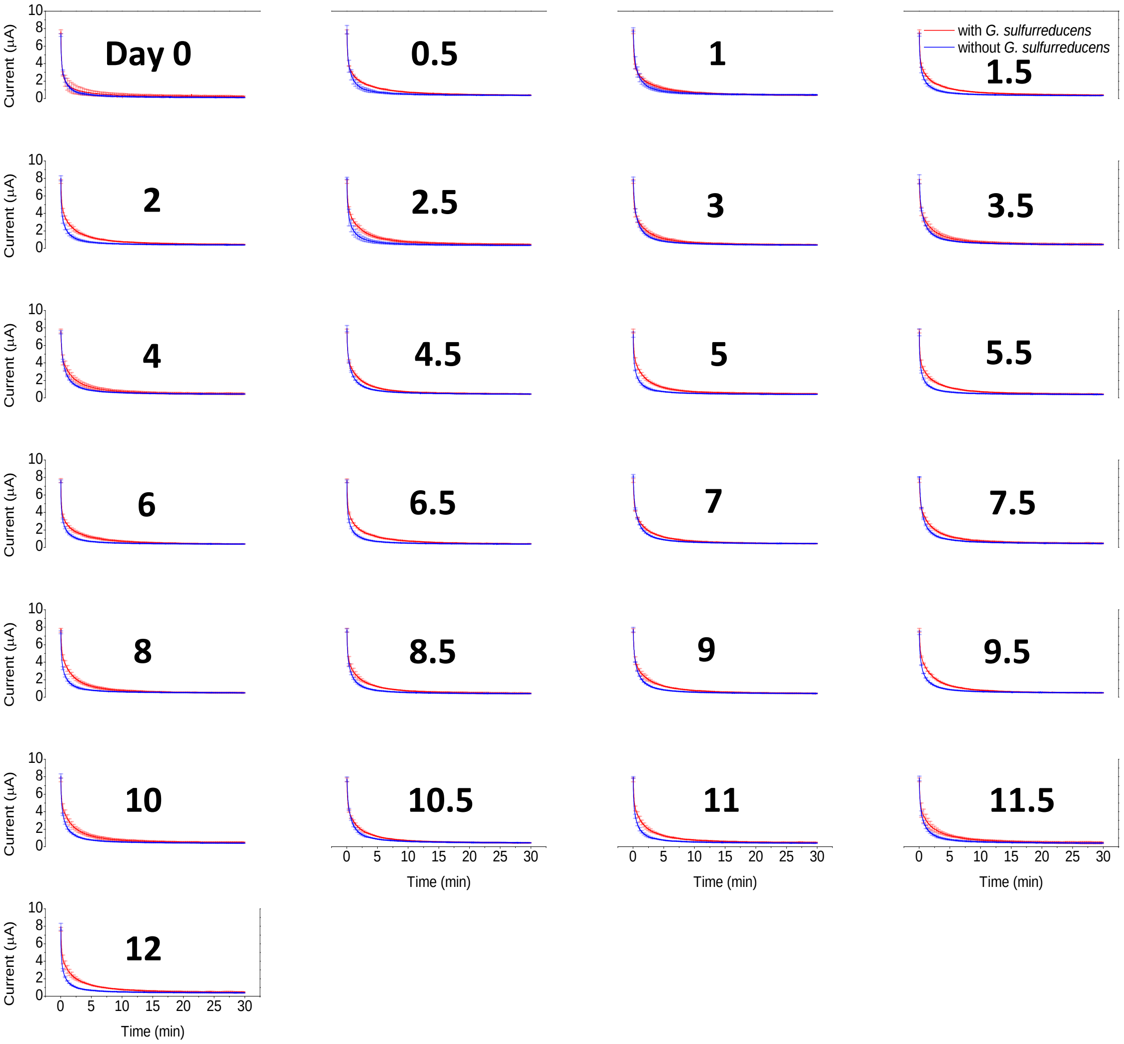

Figure S10 | Full discharging current profile of the capacitive electron transfer through the pyrogenic carbon matrices that was produced at 500°C during the bioelectrochemical pure-culture incubation (32°C and ambient light). A control incubation without *G. sulfurreduccens* was also performed to confirm that the increased discharging current through the carbon matrices was a result of the alternative respiration of *G. sulfurreduccens* by using the capacitive electron transfer as an electron snorkeling mechanism. The discharging current was recorded every half a day and the discharging duration was 30 min. The recording frequency of the discharging current was 0.1 s^{-1}. Error bars are s.d. of triplicate measurements (N=3). Data are presented as mean values +/- s.d.

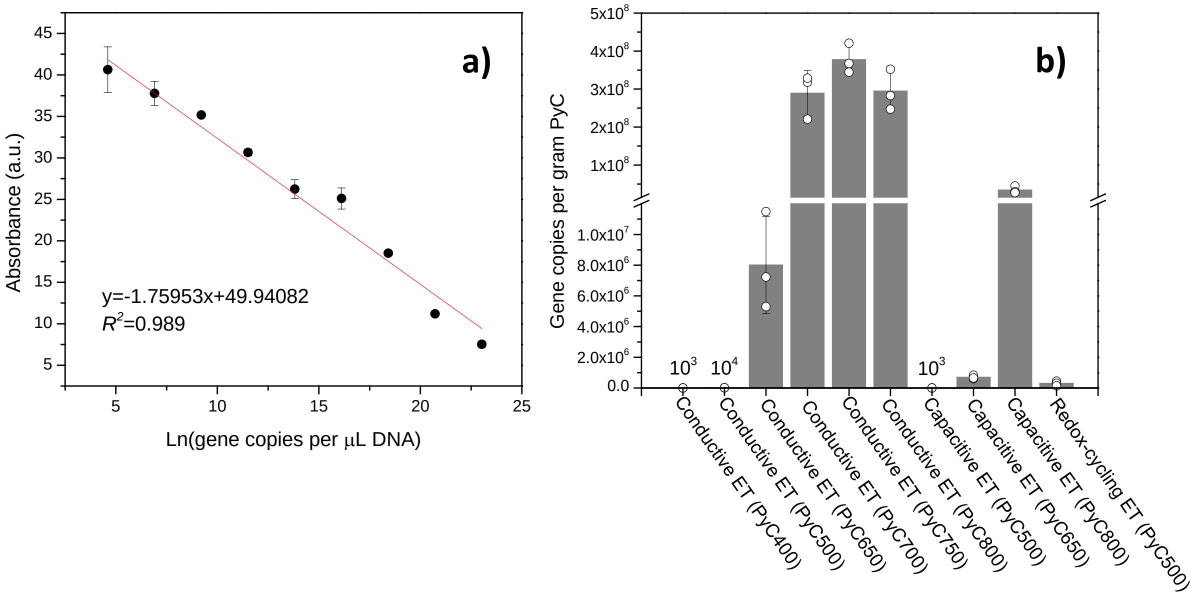

Figure S11 | Quantification of *G. sulfurreducens* biomass that respires on pyrogenic carbon during microcosm and bioelectrochemical pure-culture incubations (32°C and ambient light). a. Standard curve. **b.** Copy numbers of 16S rRNA genes of *G. sulfurreducens* respiring on pyrogenic carbon through the redox-cycling electron transfer (ET) of the functional groups and the capacitive and conductive ET of the carbon matrices. Open circles indicate the corresponding data points of the bar chart. Error bars are s.d. of triplicate measurements (N=3). Data are presented as mean values +/- s.d.

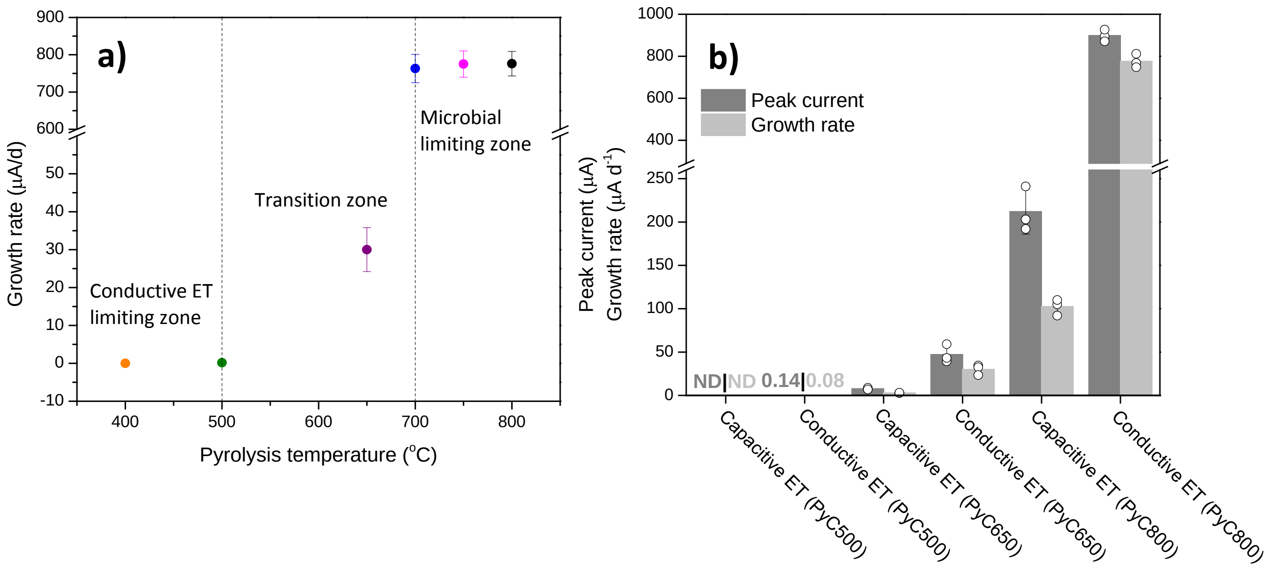

Figure S12 | Comparison of electron transfer kinetics of the pyrogenic carbon matrices in the bioelectrochemical pure-culture incubations (32°C and ambient light). a. Electron transfer kinetics of the conductive electron transfer (ET) in sustaining the alternative respiration of *G. sulfurreducens*. The electron transfer kinetics was expressed by the growth rate of *G. sulfurreducens*, which was obtained by the linear fit of the growth current slope (**Figure 3c** in the main text) at the exponential phase. Three kinetic zones had been identified. First was the conductive electron transfer limiting zone (at low pyrolysis temperature range) in which the electron snorkeling was controlled by the conductive electron transfer through carbon matrices; second was the transition zone (at intermediate pyrolysis temperature range); and third was the microbial limiting zone (at high pyrolysis temperature range) in which the electron snorkeling was limited by microbial respiration. That is, even though the conductive electron transfer rate constant still significantly increased about 4 times from 4.8×10^{-3} to 18×10^{-3} cm s^{-1} from the pyrogenic carbon produced at 700°C to that produced at 800°C[24], the electron snorkeling rate remained constant. **b.** Comparison of electron transfer kinetics between the capacitive and conductive ET in sustaining the alternative respiration of *G. sulfurreducens*. The comparison was made based on both current peak and current slope at the exponential phase (i.e., growth rate) of the growth current (**Figure 3b** and **c** in the main text) of *G. sulfurreducens*. Open circles indicate the corresponding data points of the bar chart. Error bars are s.d. of triplicate measurements (N=3). Data are presented as mean values +/- s.d.

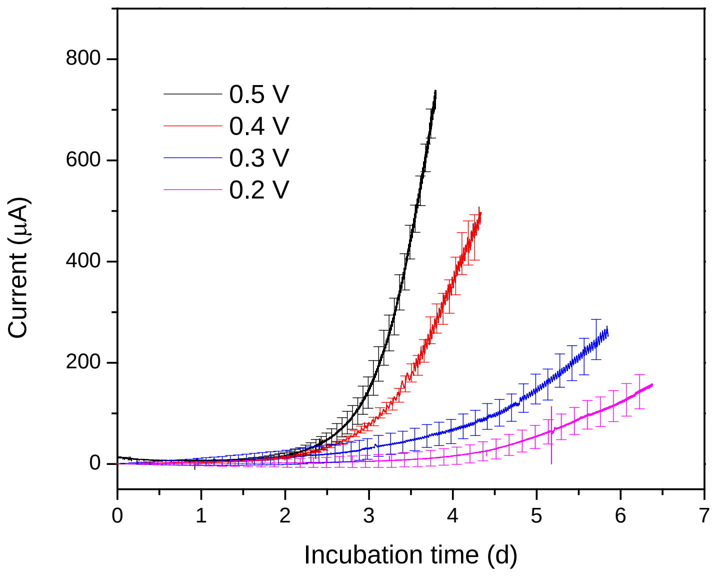

Figure S13 | Growth current of *G. sulfurreducens* supported by the conductive electron transfer of the carbon matrices during the bioelectrochemical pure-culture incubations (32°C and ambient light). The growth current was obtained at low to high (+0.2 to +0.5 V vs. standard hydrogen electrode) terminal electron accepting potentials to estimate the dependency of the electron transfer kinetics on terminal electron acceptors. Error bars are s.d. of triplicate measurements (N=3). Data are presented as mean values +/- s.d.

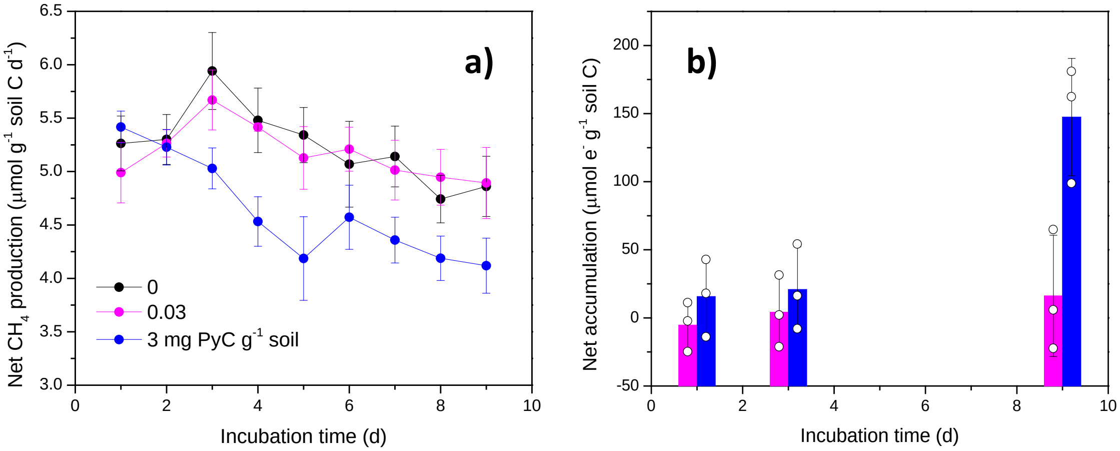

Figure S14 | Effect of pyrogenic carbon accumulation on CH_4 suppression (a) and electron accumulation to support alternative respiration in the peat soil (incubated in the dark at 32°C) (b). The chart legend in **a** indicates the concentration of pyrogenic carbon, which increased from 0 to 0.03 to 3 mg pyrogenic carbon (PyC) per gram wet soil. The chart legend in **a** also applies to chart **b.** The net electron accumulation was obtained by setting the electron accumulation in the pyrogenic carbon-free peat soil (i.e., 0 mg PyC g^{-1} soil) as background. Open circles indicate the corresponding data points of the bar chart. Error bars are s.d. of triplicate measurements (N=3). Data are presented as mean values +/- s.d.

Supplementary Tables

Table S1 | $\delta^{13}C$ (‰) signatures of the gas phase in microcosm peat-soil incubations

Day	$\delta^{13}CO_2$, Control	$\delta^{13}CO_2$, PyC500-3 mg g-1	$\delta^{13}CO_2$, PyC400-3 mg g-1	$\delta^{13}CH_4$, Control	$\delta^{13}CH_4$, PyC500-3 mg g-1	$\delta^{13}CH_4$, PyC400-3 mg g-1
1	-33.2±1.7	-20.7±3.8	-19.5±1.1	-32.5±3.7	-28.1±2.8	-25.1±2.8
2	-34.7±0.5	-22.8±2.5	-24.7±0.4	-33.1±2.7	-27.6±3.4	-30.4±3.5
3	-34.5±0.7	-23.9±2.9	-25.8±0.6	-36.9±2.6	-33.8±3.6	-28.6±2.1
4	-34.0±0.6	-24.6±2.8	-27.3±0.2	-36.7±2.5	-28.9±5.5	-29.2±1.4
5	-32.7±0.4	-24.2±3.1	-27.1±0.6	-31.5±2.1	-29.0±2.8	-29.5±2.1
6	-32.6±2.3	-26.3±4.2	-26.6±0.9	-38.4±6.2	-33.1±4.4	-32.3±3.3
7	-34.5±0.7	-27.2±1.3	-26.9±0.8	-38.1±5.7	-33.0±1.4	-37.5±3.5
8	-34.0±0.2	-28.5±3.5	-26.7±0.6	-33.5±3.3	-34.6±2.0	-37.4±3.6
9	-33.4±0.6	-29.5±2.1	-26.5±2.2	-38.6±2.1	-35.7±1.8	-38.7±4.9

Table S2 | δ^{13}C (‰) signatures of the gas phase in bioelectrochemical peat-soil incubations

Day	δ$^{13}CO_2$, Control	δ$^{13}CO_2$, Conductive ET	δ$^{13}CO_2$, Capacitive ET	δ$^{13}CH_4$, Control	δ$^{13}CH_4$, Conductive ET	δ$^{13}CH_4$, Capacitive ET
1	-34.7±1.2	-34.7±0.4	-36.8±1.0	-44.8±3.6	-39.1±3.5	-44.5±2.1
2	-37.1±0.6	-35.4±0.4	-36.7±0.7	-40.3±4.1	-35.3±4.3	-41.1±4.2
3	-37.2±0.4	-41.1±4.3	-36.3±0.4	-36.5±2.2	-38.2±4.1	-40.5±6.4
4	-38.3±1.8	-35.1±4.7	-35.9±0.7	-42.6±1.1	-37.4±0.8	-34.3±4.6
5	-37.7±2.2	-35.6±0.8	-36.7±0.1	-45.5±7.8	-37.5±0.2	-39.5±4.9
6	-35.6±3.5	-35.2±1.0	-38.0±0.4	-38.3±1.3	-36.8±5.3	-43.1±2.8
7	-35.8±3.1	-36.7±0.1	-35.8±2.3	-39.2±2.6	-44.4±3.4	-39.5±3.5
8	-35.1±0.4	-36.6±0.8	-36.6±0.1	-38.1±5.8	-47.5±3.5	-40.5±4.2
9	-36.5±1.6	-40.2±4.9	-37.1±2.5	-41.8±4.2	-35.5±0.7	-36.5±4.9

References

1 Sun, C., Brauer, S., Cadillo Quiroz, H., Zinder, S. & Yavitt, J. Seasonal changes in methanogenesis and methanogenic community in three peatlands, New York State. *Front. Microbiol.* **3** (2012).

2 Bräuer, S. L., Yavitt, J. B. & Zinder, S. H. Methanogenesis in McLean Bog, an acidic peat bog in Upstate New York: Stimulation by H_2/CO_2 in the presence of rifampicin, or by low concentrations of acetate. *Geomicrobiol. J.* **21**, 433-443 (2004).

3 Yavitt, J. B. & Seidman-Zager, M. Methanogenic conditions in northern peat soils. *Geomicrobiol. J.* **23**, 119-127 (2006).

4 Bushnell, B. BBMap short-read aligner, and other bioinformatics tools. *University of California, Berkeley, CA* (2015).

5 Masella, A. P., Bartram, A. K., Truszkowski, J. M., Brown, D. G. & Neufeld, J. D. PANDAseq: paired-end assembler for illumina sequences. *BMC bioinformatics* **13**, 31 (2012).

6 Caporaso, J. G. *et al.* QIIME allows analysis of high-throughput community sequencing data. *Nature methods* **7**, 335 (2010).

7 Edgar, R. C. Search and clustering orders of magnitude faster than BLAST. *Bioinformatics* **26**, 2460-2461 (2010).

8 Harbison, A. B., Carson, M. A., Lamit, L. J., Basiliko, N. & Bräuer, S. L. A novel isolate and widespread abundance of the candidate alphaproteobacterial order (Ellin 329), in southern Appalachian peatlands. *FEMS Microbiol. Lett.* **363** (2016).

9 Cloutier, M. L. C. *Microbial community analysis coupled with geochemical studies reveal factors affecting biotic Mn(II) oxidation in situ* Master thesis, Appalachian State University, (2016).

10 DeSantis, T. Z. *et al.* Greengenes, a chimera-checked 16S rRNA gene database and workbench compatible with ARB. *Appl. Environ. Microbiol.* **72**, 5069-5072 (2006).

11 Yavitt, J. B., Williams, C. J. & Wieder, R. K. Production of methane and carbon dioxide in peatland ecosystems across North America: Effects of temperature, aeration, and organic chemistry of peat. *Geomicrobiol. J.* **14**, 299-316 (1997).

12 Bridgham, S. D., Updegraff, K. & Pastor, J. Carbon, nitrogen, and phosphorus mineralization in northern wetlands. *Ecology* **79**, 1545-1561 (1998).

13 Glatzel, S., Basiliko, N. & Moore, T. Carbon dioxide and methane production potentials of peats from natural, harvested and restored sites, eastern Québec, Canada. *Wetlands* **24**, 261-267 (2004).

14 Roden, E. E. & Wetzel, R. G. Organic carbon oxidation and suppression of methane production by microbial Fe(III) oxide reduction in vegetated and unvegetated freshwater wetland sediments. *Limnol. Oceanogr.* **41**, 1733-1748 (1996).

15 Duddleston, K. N., Kinney, M. A., Kiene, R. P. & Hines, M. E. Anaerobic microbial biogeochemistry in a northern bog: Acetate as a dominant metabolic end product. *Global Biogeochem. Cycles* **16**, 11-11-11-19 (2002).

16 Sivan, O., Antler, G., Turchyn, A. V., Marlow, J. J. & Orphan, V. J. Iron oxides stimulate sulfate-driven anaerobic methane oxidation in seeps. *PNAS* **111**, E4139-E4147 (2014).

17 Walpen, N., Getzinger, G. J., Schroth, M. H. & Sander, M. Electron-donating phenolic and electron-accepting quinone moieties in peat dissolved organic matter: Quantities and redox

transformations in the context of peat biogeochemistry. *Environ. Sci. Technol.* **52**, 5236-5245 (2018).
18 Cory, R. M. & McKnight, D. M. Fluorescence spectroscopy reveals ubiquitous presence of oxidized and reduced quinones in dissolved organic matter. *Environ. Sci. Technol.* **39**, 8142-8149 (2005).
19 Roden, E. E. *et al.* Extracellular electron transfer through microbial reduction of solid-phase humic substances. *Nat. Geosci.* **3**, 417-421 (2010).
20 Klüpfel, L., Piepenbrock, A., Kappler, A. & Sander, M. Humic substances as fully regenerable electron acceptors in recurrently anoxic environments. *Nat. Geosci.* **7**, 195-200 (2014).
21 Gao, C., Sander, M., Agethen, S. & Knorr, K.-H. Electron accepting capacity of dissolved and particulate organic matter control CO2 and CH4 formation in peat soils. *Geochim. Cosmochim. Acta* **245**, 266-277 (2019).
22 Lau, M., Sander, M., Gelbrecht, J. & Hupfer, M. Solid phases as important electron acceptors in freshwater organic sediments. *Biogeochemistry* **123**, 49-61 (2015).
23 DeCiucies, S., Whitman, T., Woolf, D., Enders, A. & Lehmann, J. Priming mechanisms with additions of pyrogenic organic matter to soil. *Geochim. Cosmochim. Acta* **238**, 329-342 (2018).
24 Sun, T. *et al.* Rapid electron transfer by the carbon matrix in natural pyrogenic carbon. *Nat. Commun.* **8**, 14873 (2017).
25 Wu, S. *et al.* Redox-active oxygen-containing functional groups in activated carbon facilitate microbial reduction of ferrihydrite. *Environ. Sci. Technol.* **51**, 9709-9717 (2017).
26 Xu, S. *et al.* Biochar-facilitated microbial reduction of hematite. *Environ. Sci. Technol.* **50**, 2389-2395 (2016).
27 Saquing, J. M., Yu, Y.-H. & Chiu, P. C. Wood-derived black carbon (Biochar) as a microbial electron donor and acceptor. *Environ. Sci. Technol. Lett.* **3**, 62-66 (2016).
28 Prévoteau, A., Ronsse, F., Cid, I., Boeckx, P. & Rabaey, K. The electron donating capacity of biochar is dramatically underestimated. *Sci. Rep.* **6**, 32870 (2016).
29 Aeschbacher, M., Vergari, D., Schwarzenbach, R. P. & Sander, M. Electrochemical analysis of proton and electron transfer equilibria of the reducible moieties in humic acids. *Environ. Sci. Technol.* **45**, 8385-8394 (2011).
30 Khodadad, C. L. M., Zimmerman, A. R., Green, S. J., Uthandi, S. & Foster, J. S. Taxa-specific changes in soil microbial community composition induced by pyrogenic carbon amendments. *Soil Biol. Biochem.* **43**, 385-392 (2011).
31 Feng, Y., Xu, Y., Yu, Y., Xie, Z. & Lin, X. Mechanisms of biochar decreasing methane emission from Chinese paddy soils. *Soil Biol. Biochem.* **46**, 80-88 (2012).
32 Klüpfel, L., Keiluweit, M., Kleber, M. & Sander, M. Redox properties of plant biomass-derived black carbon (Biochar). *Environ. Sci. Technol.* **48**, 5601-5611 (2014).
33 Marsili, E., Rollefson, J. B., Baron, D. B., Hozalski, R. M. & Bond, D. R. Microbial biofilm voltammetry: Direct electrochemical characterization of catalytic electrode-attached biofilms. *Appl. Environ. Microbiol.* **74**, 7329-7337 (2008).
34 Guzman, J. J. L., Pehlivaner Kara, M. O., Frey, M. W. & Angenent, L. T. Performance of electro-spun carbon nanofiber electrodes with conductive poly(3,4-ethylenedioxythiophene) coatings in bioelectrochemical systems. *J. Power Sources* **356**, 331-337 (2017).

35 Aeschbacher, M., Sander, M. & Schwarzenbach, R. P. Novel electrochemical approach to assess the redox properties of humic substances. *Environ. Sci. Technol.* **44**, 87-93 (2010).
