# Supplementary material for: Suppressing peatland methane production by electron snorkeling through pyrogenic carbon in controlled laboratory incubations
Source: Nat Commun. 2021 Jul 5;12:4119. doi: 10.1038/s41467-021-24350-y (PMC8257765; doi:10.1038/s41467-021-24350-y)
Supplement: Supplementary file 3 — Description of additional supplementary files [file 41467_2021_24350_MOESM3_ESM.docx]

Description of additional supplementary information

Title: Dataset 1

Description: **Source data of Figure 1, Figure 3a-b, and Supplementary Figures S2, S4, S6, S7, S8, and S9.**

Title: Dataset 2

Description: **Source data of Figure 3c-d and Supplementary Figures S10 and S11.**

Title: Dataset 3

Description: **Source data of Figure 4 and Supplementary Figures S5, S12, and S13.**

Title: Dataset 4

Description: **Source data of Supplementary Figure S1.**
