# Supplementary material for: Suppressing peatland methane production by electron snorkeling through pyrogenic carbon in controlled laboratory incubations
Source: Nat Commun. 2021 Jul 5;12:4119. doi: 10.1038/s41467-021-24350-y (PMC8257765; doi:10.1038/s41467-021-24350-y)
Supplement: Supplementary file 8 — Reporting Summary [file 41467_2021_24350_MOESM8_ESM.pdf]

nature research

Corresponding author(s): Largus T. Angenent

Last updated by author(s): May 20, 2021

Reporting Summary

Nature Research wishes to improve the reproducibility of the work that we publish. This form provides structure for consistency and transparency in reporting. For further information on Nature Research policies, see our Editorial Policies and the Editorial Policy Checklist.

Statistics

For all statistical analyses, confirm that the following items are present in the figure legend, table legend, main text, or Methods section.

n/a	Confirmed	
☐	☒	The exact sample size (*n*) for each experimental group/condition, given as a discrete number and unit of measurement
☐	☒	A statement on whether measurements were taken from distinct samples or whether the same sample was measured repeatedly
☐	☒	The statistical test(s) used AND whether they are one- or two-sided<br>*Only common tests should be described solely by name; describe more complex techniques in the Methods section.*
☐	☒	A description of all covariates tested
☐	☒	A description of any assumptions or corrections, such as tests of normality and adjustment for multiple comparisons
☐	☒	A full description of the statistical parameters including central tendency (e.g. means) or other basic estimates (e.g. regression coefficient) AND variation (e.g. standard deviation) or associated estimates of uncertainty (e.g. confidence intervals)
☐	☒	For null hypothesis testing, the test statistic (e.g. *F*, *t*, *r*) with confidence intervals, effect sizes, degrees of freedom and *P* value noted<br>*Give P values as exact values whenever suitable.*
☒	☐	For Bayesian analysis, information on the choice of priors and Markov chain Monte Carlo settings
☒	☐	For hierarchical and complex designs, identification of the appropriate level for tests and full reporting of outcomes
☒	☐	Estimates of effect sizes (e.g. Cohen's *d*, Pearson's *r*), indicating how they were calculated

Our web collection on statistics for biologists contains articles on many of the points above.

Software and code

Policy information about availability of computer code

Data collection	All data were collected either by direct measurement or indirect calculation. All used calculating equations were given in Supplementary Information e.q. S1-S9.
Data analysis	All data were analyzed by Excel, Origin version 8.5, QIIME version 1.9.1, PANDAseq version 2.1, BBmp (version number not available), and USEARCH version 8

For manuscripts utilizing custom algorithms or software that are central to the research but not yet described in published literature, software must be made available to editors and reviewers. We strongly encourage code deposition in a community repository (e.g. GitHub). See the Nature Research guidelines for submitting code & software for further information.

Data

Policy information about availability of data

All manuscripts must include a data availability statement. This statement should provide the following information, where applicable:

- Accession codes, unique identifiers, or web links for publicly available datasets
- A list of figures that have associated raw data
- A description of any restrictions on data availability

The data supporting the findings of this study are available within the article and its Supplementary Information files. Source data for figures are provided with this paper.

Field-specific reporting

Please select the one below that is the best fit for your research. If you are not sure, read the appropriate sections before making your selection.

☐ Life sciences ☐ Behavioural & social sciences ☒ Ecological, evolutionary & environmental sciences

For a reference copy of the document with all sections, see nature.com/documents/nr-reporting-summary-flat.pdf

Ecological, evolutionary & environmental sciences study design

All studies must disclose on these points even when the disclosure is negative.

Study description	In this study, we show that accumulation of pyrogenic carbon can suppress post-fire methane production in northern peatlands, which indirectly but positively buffers and rebalances the fire-derived greenhouse gas emissions. To deepen our understanding of the suppressing mechanisms, we conducted over 100 microcosm and bioelectrochemical incubations that were equipped with a state-of-the-art interfacial electron transfer detection system and a real-time gas and isotopic analyzer. We found that the accumulation of pyrogenic carbon redirected electron fluxes and facilitated alternative microbial respiration in anaerobic peat soils.
Research sample	The peat soil samples were collected at the Mclean Bog located in Dryden, New York (42°30′ N, 76°30′ W). McLean Bog is an ombrotrophic peat site, which has an area of 0.004-km2. Total peat depth is 8 m. All soil samples are re-accessable. The Mclean Bog is a national natural landmark with enriched organic carbon content and known methanogenesis activity and CH_4 production. It is, therefore, a suitable location for the study of the interaction between methanogenesis and carbon electron transfer.
Sampling strategy	The peat soil samples were collected in June at the Mclean Bog. Soils were sampled at several bog locations, spreading from the center to the near-edge areas. We sampled soils by hand scooping. The sampling depth reached approximately 10-15 cm below the water level. We used two layers of Ziplock bags to store each pack of soil samples to prevent the oxygen penetration during transportation. The soils were stored in the lab at room temperature and dark environment until the incubation started. We did not perform predetermination of sample size. We sampled the soils at well-spread locations to ensure the representativeness of the physicochemical, biogeochemical, and metabolical conditions of the Mclean Bog.
Data collection	Electrochemical data were collected by potentiostat (Bio-Logic model VSP) and recorded by its built-in software (EC-Lab). Gas and isotopic data were collected by stable isotope analyzer (Picarro model G2201-I) and recorded by its built-in software (CRDS Data Viewer). Sequences were collected on the MiSeq platform by the Department of Energy's (DOE) Joint Genome Institute (JGI) for bacteria and archaea using the V4 region of SSU rRNA (515/806). Raw sequence data was obtained from the JGI database. TS, JJG, and JS were responsible for data collection.
Timing and spatial scale	We collected data in lab incubations. We don't have field site data collection. Pure-culture incubation started at January, 2017 and stopped at June, 2017. Peat-soil incubation started at June, 2017 and stopped at November, 2017. There was no time gap for incubation. We started the peat-soil incubations as early as possible after the soil samples were collected to prevent property modification of the peat soil. The criteria of stopping incubation was the exhaustion of substrate, that is, the considerable drop of CO_2 production. The Mclean Bog has an area of 0.004-km2 and therefore the collected data reflected a spatial scale of this area.
Data exclusions	No data were excluded from the analyses.
Reproducibility	We guaranteed reproducibility by always setting up incubations in triplicates and all results from each replicate were reported.
Randomization	The incubations were grouped by microbial community composition (i.e., pure-culture or native soil community) and incubation techniques (i.e., bioelectrochemical or microcosm incubation).
Blinding	We made our experiment as blind as possible by eliminating artificial data reading and only rely on instrument data recording

Did the study involve field work? ☒ Yes ☐ No

Field work, collection and transport

Field conditions	The peat soil samples were collected at the Mclean Bog. Mclean Bog is an ombrotrophic peat site, which has an area of 0.004-km2. The bog contains no in-let or out-let stream. Mean annual precipitation is 932 mm and mean annual temperature is 7.8 degree Celsius. It has a continuous cover of Sphagnum mosses (S.magellanicum, S. angustifolium) with scattered patches of ericaceous shrubs (Chamaedaphne calyculata) and common cottongrass (Eriophorum angustifolium). The 8m deep peat is acidic with a pH ranging from 3.4 to 4.2. The peat is saturated to close to the peat surface, except during extreme drought periods.
Location	Mclean Bog is located in Dryden, New York (42°30′ N, 76°30′ W). Mclean Bog has an elevation of 335 m and a depth of 8 m.
Access & import/export	Mclean Bog is administratively operated by Cornell Plantation. As Cornell employees, we have full access to this site.
Disturbance	Disturbance of the site is minor as we sampled the soil in a very scattered way and the total amount of sampled soil was less than 5 kg. We believe the peat body was still intact.

Reporting for specific materials, systems and methods

We require information from authors about some types of materials, experimental systems and methods used in many studies. Here, indicate whether each material, system or method listed is relevant to your study. If you are not sure if a list item applies to your research, read the appropriate section before selecting a response.

Materials & experimental systems

n/a	Involved in the study
☒	☐ Antibodies
☒	☐ Eukaryotic cell lines
☒	☐ Palaeontology and archaeology
☒	☐ Animals and other organisms
☒	☐ Human research participants
☒	☐ Clinical data
☒	☐ Dual use research of concern

Methods

n/a	Involved in the study
☒	☐ ChIP-seq
☒	☐ Flow cytometry
☒	☐ MRI-based neuroimaging
